# A Collaborative Filtering Approach for Protein-Protein Docking Scoring Functions

**DOI:** 10.1371/journal.pone.0018541

**Published:** 2011-04-22

**Authors:** Thomas Bourquard, Julie Bernauer, Jérôme Azé, Anne Poupon

**Affiliations:** 1 Bioinformatics Group, INRIA AMIB, Laboratoire de Recherche en Informatique, Université Paris-Sud, Orsay, France; 2 Bioinformatics Group, INRIA AMIB, Laboratoire d'Informatique (LIX), École Polytechnique, Palaiseau, France; 3 INRIA Nancy Grand Est, LORIA, Vandoeuvre-lès-Nancy, France; 4 BIOS Group, INRA, UMR85, Unité Physiologie de la Reproduction et des Comportements, Nouzilly, France; 5 CNRS, UMR6175, Nouzilly, France; 6 Université François Rabelais, Tours, France; Miami University, United States of America

## Abstract

A protein-protein docking procedure traditionally consists in two successive tasks: a search algorithm generates a large number of candidate conformations mimicking the complex existing *in vivo* between two proteins, and a scoring function is used to rank them in order to extract a native-like one. We have already shown that using Voronoi constructions and a well chosen set of parameters, an accurate scoring function could be designed and optimized. However to be able to perform large-scale *in silico* exploration of the interactome, a near-native solution has to be found in the ten best-ranked solutions. This cannot yet be guaranteed by any of the existing scoring functions.

In this work, we introduce a new procedure for conformation ranking. We previously developed a set of scoring functions where learning was performed using a genetic algorithm. These functions were used to assign a rank to each possible conformation. We now have a refined rank using different classifiers (decision trees, rules and support vector machines) in a collaborative filtering scheme. The scoring function newly obtained is evaluated using 10 fold cross-validation, and compared to the functions obtained using either genetic algorithms or collaborative filtering taken separately.

This new approach was successfully applied to the CAPRI scoring ensembles. We show that for 10 targets out of 12, we are able to find a near-native conformation in the 10 best ranked solutions. Moreover, for 6 of them, the near-native conformation selected is of high accuracy. Finally, we show that this function dramatically enriches the 100 best-ranking conformations in near-native structures.

## Introduction

Most proteins fulfill their functions through the interaction with other proteins [1]. The interactome appears increasingly complex as the experimental means used for its exploration gain in precision [2]. Although structural genomics specially addressing the question of 3D structure determination of protein-protein complexes have led to great progress, the low stability of most complexes precludes high-resolution structure determination by either crystallography or NMR. 3D structure of complexes are thus poorly represented in the Protein Data Bank (PDB) [3]. The fast and accurate prediction of the assembly from the structures of the individual partners, called protein-protein docking, is therefore of great value [4]. However, available docking procedures technically suitable for large-scale exploration of the proteome have shown their limits [5], [6]. Indeed, amongst the easily available methods for such exploration, a near-native solution is found in the 10 best-ranked solutions (top 10) only for 34% of the studied complexes. For biologists, exploring 10 different conformations for experimental validation is already a huge effort. Making this exploration knowing that the prediction will be confirmed only in one case out of three is completely unacceptable. Consequently, large-scale protein-protein docking will be useful for biologists only if a near-native solution can be found in the top 10 in almost all cases (ideally in the top 5 or even the top 3).

A docking procedure consists in two tasks, generally consecutive and largely independent. The first one, called exploration, consists in building a large number of candidates by sampling the different possible orientations of one partner relatively to the other. The second task consists in ranking the candidates using a scoring function in order to extract near-native conformations. To be accurate, scoring functions have to take into account both the geometric complementarity and the physico-chemistry of amino acids in interaction, since they both contribute to the stability of the assembly [7], [8].

Modeling multi-component assemblies often involves computationally expensive techniques, and exploring all the solutions is often not feasible. Consequently, we previously introduced a coarse-grained model for protein structure based on the Voronoi tessellation. This model allowed the set up of a method for discriminating between biological and crystallographic dimers [9], and the design of an optimized scoring function for protein-protein docking [10], [11]. These results show that this representation retains the main properties of proteins and proteins assemblies 3D structures, making it a precious tool for building fast and accurate scoring methods. We have also explored the possibility to use a power diagram or Laguerre tessellation model, which gives a more realistic representation of the structure. However we have shown that this model does not give better results and increases algorithmic complexity [12].

In this study, using the Voronoi representation of protein structure, and an in-lab conformation generation algorithm, we show different ways of optimizing the scoring method based on probabilistic multi-classifiers adaptation and genetic algorithm.

## Methods

### Structure Representation and Conformation Generation

**Figure 1:**
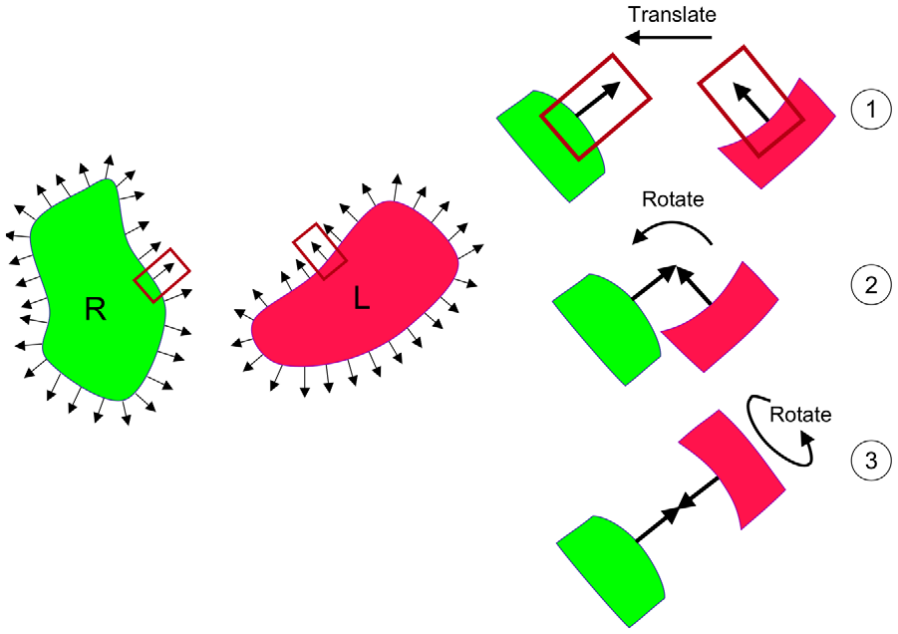
Conformation generation method.

Like in our previous work [9]–[12], a coarse-grain model is used to represent the protein structure. We define a single node for each residue (the geometric center of side chain, including C*_a_*), the Delaunay triangulation (dual of the Voronoi diagram) of each partner is then computed using CGAL [13] and the Voronoi tessellation is built. The generation of candidate conformations is performed as follows. For each node, a pseudo-normal vector is built by summing the vectors linking this node to its neighbors. In non-convex regions, this vector might point towards the interior of the protein. In this case the opposite vector is taken. Depending on the amino acid type, the length of this vector is made equal to the radius of a sphere whose volume is equal to the average volume occupied by this type of amino acid. This mean volume is taken from Pontius *et al.* [14]. For each possible pair of vectors (one in each partner), one of the vectors is translated so as to bring its extremity on the extremity of the first vector (step 1 on Figure 1). The second partner is then rotated so as to oppose the two vectors (step 2 on Figure 1). The second partner is then rotated around this new axis (step 3 on Figure 1), and a conformation of the complex is build every 5° rotation.

Although not all degrees of freedom are considered (the two normal vectors are always aligned in our method, but we could have considered varying the angle between them), we obtain a near-native conformation for all the complexes in the learning set.

### Learning set

Our positive examples set is composed of native structures. We complemented our previous set [12] with the reference set from [15]. This set contains 211 bound-unbound and unbound-unbound complexes (complexes for which the 3D structure of at least one partner is known). SCOP [16] was used to remove redundancy (for two complexes AB and CD, if A and C belong to the same SCOP family, and B and D also belong to the same family, the complex is eliminated).

Negative examples (decoys, or non-native conformations) were generated by applying the previously described generation method to each complex of our native structures set. Only conformations having a minimal interface area of 400 Å^2^ and a root mean square deviation (RMSD) relative to the native conformation higher than 10 Å were retained. Within this ensemble, 15 non-native conformations were chosen for each native conformations, resulting in 2980 negative examples in the learning set. As observed in our previous studies, missing values are a serious issue for scoring function optimization. All the non-native conformations presenting a too high number of missing values were removed. This number was taken to be twice the number of missing values in the corresponding native structure. 20 such non-native conformations for each native structure were randomly chosen from the initial decoys set.

### Training Parameters

The coarse-grained Voronoi tessellation allows simple description of the protein-protein interface. 96 training attributes [12] based on properties of residues and pairs present at the interface have been used. For pair parameters, residues were binned in six categories: hydrophobic (ILVM), aromatics (FYW), small (AGSTCP), polar (NQ), positive (HKR) and negative (DE). These categories are also used to compute the 12 last parameters. Retained parameters are:

- *c*_1_: The Voronoi interface area.
- *c*_2_: The total number of interface residues.
- *c*_3_ to *c*_22_: The fraction of each type of interface residues.
- *c*_23_ to *c*_42_: The mean volume of Voronoi cells for the interface residues.
- *c*_43_ to *c*_63_: The fraction of pairs of interface residues.
- *c*_64_ to *c*_84_: The mean node-node distance in pairs of interface residues.
- *c*_85_ to *c*_90_: The fraction of interface residues for each category.
- *c*_91_ to *c*_96_: The mean volume of Voronoi cells for the interface residues for each category.

All parameters were computed on the complete interface, defined as all the residues having at least one neighbor belonging to the second partner, including residues in contact with solvent molecules.

### Genetic algorithm

Using previously defined training attributes, genetic algorithms are used to find family of functions that optimize the ROC (Receiver Operating Characteristics) criterion. We used a 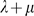 scheme, with 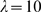 parents and 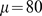 children, and a maximum of 500 generations. We used a classical cross-over and auto-adaptative mutations. The ROC criterion is commonly used to evaluate the performance of learning procedures by measuring the area under the ROC curve (AUC). The ROC curve is obtained by plotting the proportion of true positives against false positives.

The scoring functions used in this work have the form:
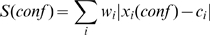
(1)where 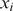 is the value of parameter 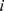 and 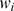 and 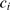 are the weights and centering values respectively for parameter 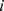. 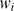 and 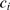 are optimized by the learning procedure. Learning was performed in a 10-fold cross-validation setting. Ten groups of models were randomly chosen, each excluding 10% of the training set. Learning was repeated 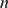 times for each training subset. Consequently, each conformation is evaluated using 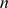 different scoring functions, and for final ranking, the sum of the ranks obtained by each function is used.

As described in the Results section, the number of functions 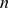 used in the computation of the final rank might have an impact on the quality of the global ranking.

### Collaborative filtering methods

Several previous studies have shown the strength of Collaborative Filtering (CF) techniques in Information Retrieval problems [17] to increase the accuracy of the prediction rate. In a common CF recommender system, there is a list of 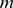 users, 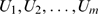 and a list of 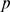 items, 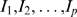 and each user gives a mark to each object. This mark can also be inferred from the user's behaviour. The final mark of each object is then defined by the ensemble of marks received from each user.

In the present work, a classifier is a user, and conformations are the items. Each classifier (user) assigns to each item (conformation) a binary label (or mark): *native'* (+) or *non native* (−).

12 classifiers have been trained on the learning set (see “Results and Discussion”), deriving from four different methods: decision trees, rules, logistic regression and SVM (Support Vector Machine). Most optimizations were done using Weka [18]. The *SVMlight* [19] software was used for SVM computations.

In a first approach, we have used a default voting system: the conformations are ranked according to the number of + marks they have received. Since we have 12 classifiers, this determines 13 different categories: 12+, 11+, …, 0+.

Because 13 categories is far from enough to efficiently ranks a very large number of conformations, we have also used a second approach using an amplification average voting system. In this system, the votes of each classifier are weighted by the precision. Consequently, the + vote of each classifier is different from the + vote of a different classifier. This results in 2^12^ categories. The categories are ordered according to:
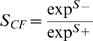
(2)


Where 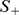 (respectively 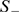) is the sum of the precisions of the classifiers that have voted + (respectively −) for conformations of this category. This score is assigned to each conformation of the considered category.
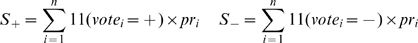
(3)


Where 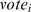 represents the vote of the 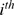 classifier and 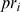 represents its precision. In a unweighted approach, 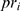 is set to 1 for all the classifiers.

Finally, the CF and GA methods have been coupled. For each conformation evaluated with at least one positive vote (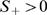), the score 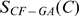 of a given conformation 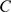 is the product of the rank obtained by 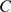 in the GA, and 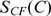. For conformations receiving only negative votes, the score 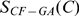 is set to be maximal. The evaluated conformations are then re-ranked according to this score (in decreasing order). It should be noted that scores (and consequently ranks) obtained through this method are not necessarily unique. To measure the number of possible ranks for each method, taking into account the number of examples to classify, we will use the granularity as defined in equation 4.
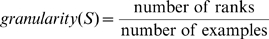
(4)


Where 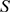 is a set of evaluated conformations.

### Evaluation of learning accuracy

The most commonly used criterion for evaluating the efficiency of a learning procedure is the Area Under the ROC curve (ROC AUC). The ROC curve is obtained by plotting the proportion of true positives against the proportion of false positives. A perfect learning should give an AUC of 1 (all the true positives are found before any of the negatives), whereas a random function has an AUC of 0.5 (each prediction has probabilities of 0.5 to be correct or incorrect).

To measure the performances of the different scoring functions we use precision, recall and accuracy using 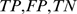 and 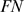 as in the confusion matrix (see Table 1). We will also use false negative rate (FNR) and true negative rate (TNR).

**Table 1:** Confusion matrix.

		solution	
		+	-
prediction	+	TP	FP
	-	FN	TN

TP: true positives, FP: false positives, FN: false negatives, TN: true negatives.

These values can be computed as:
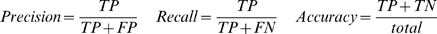


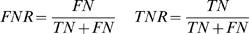



### CAPRI Experiments

To evaluate the accuracy of our CF-GA scoring procedure, we developed two protocols based on targets 22 to 40 of the CAPRI (Critical Assessment of PRedicted Interaction) experiment. CAPRI is a blind prediction experiment designed to test docking and scoring procedures [20], [21]. In the scoring experiment, a large set of models submitted by the docking predictors is made available to the community to test scoring functions independently of conformation generation algorithms.

Four targets where eliminated for different reasons:

- The structure of target 31 has not yet been released, making it impossible to evaluate the obtained results.
- The native 3D structure of target 30 is still a vexed question [22].
- Targets 33 and 34 are protein-RNA complexes and our scoring method is not adapted to this problem yet.

For each target, the scoring ensemble was evaluated using GA, CF and CF-GA methods.

For reasons exposed in “Results and Discussion”, candidate conformations where evaluated according to two different sets of criteria.

In the 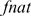 evaluation, we use only the 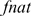 criterion, which is the fraction of native contacts (the fraction of contacts between the two partners in the evaluated conformation that do exist in the native structure). Four quality classes can be defined:

- High (*fnat* ≥0.5),
- Medium (0.3≤ *fnat* <0.5),
- Acceptable (0.1≤ *fnat* <0.3),
- Incorrect (*fnat*<0.1)

CAPRI evaluation [21], [23] also uses two other criteria: the 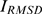 (*RMSD* between prediction and native structure computed only on interface atoms) and 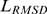 (*RMSD* computed on all the atoms of the smallest protein, the largest protein of prediction and native structure being superimposed). Again four quality classes are defined:

- High: (*fnat* ≥0.5) and (*I_RMSD_* ≤1 or *L_RMSD_* ≤1)
- Medium: [(0.3≤ *fnat* <0.5) and (*I_RMSD_* ≤2.0 or *L_RMSD_* ≤5.0)]**or** [(*fnat* >0.5 and *I_RMSD_*>1.0 or *I_RMSD_*>1.0)]
- Acceptable: [(0.1≤ *fnat* <0.3) and (*I_RMSD_* ≤4.0 or *L_RMSD_* ≤10.0)] **or** [*fnat* >0.3 and (*L_RMSD_* >5.0 or *I_RMSD_* >2.0)]
- Incorrect.

## Results and Discussion

In our previous work, we have used different flavors of genetic algorithm (GA) optimization to obtain scoring functions for protein-protein docking. Since we have reached the limits of the precision that can be obtained with GA alone, we combined the GA-based scoring function with scoring functions built using four other learning algorithms:

- Logistic regression (LR) [24];
- Support Vector Machines [25], using either radial-based function (RBF), linear kernel (LK), polynomial kernel (PK) or 2 and 4 quadratic kernels (QK2 and QK4);
- Decision trees, using the C4.5 learner [26] and, J48, its implementation in Weka [18], using 2, 5 and 10 as minimum numbers of examples required to build a leaf (classifiers J48-M2, J48-M5 and J48-M10 respectively);
- Two-rules learners, using two different implementations (JRIP [27] and PART [28]), using again 2, 5 and 10 as minimum numbers of examples required to build a rule (classifiers JRIP-M2, JRIP-M5, JRIP-M10, PART-M2, PART-M5 and PART-M10).

Here we show how these 15 classifiers can be combined, in a collaborative scheme and with the genetic algorithm procedure.

### Predictions obtained with the genetic algorithm procedure

**Figure 2:**
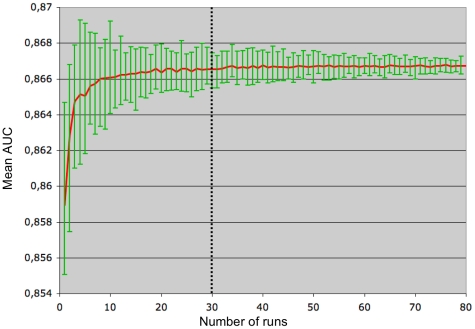
Genetic Algorithm performance as a function of the number of runs. For each number of runs 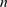, the measure of the AUC has been repeated 50 times using a 10-fold cross-validation protocol. Average, minimum and maximum values are plotted.

The sensitivity (ability to discriminate true positives from false positives, also called recall) of the genetic algorithm (GA) has been evaluated using the ROC criterion. Since GA is a heuristic, optimization must be repeated. The number of repetitions necessary for obtaining a reliable result largely depends on the specificity of the problem. To determine the number of repetitions needed in our case, we have plotted the area under the ROC curve (AUC) as a function of the number of runs. For each value of the number of runs *n*, the experiment has been repeated 50 times in 10-fold cross-validation. This allows to compute, for each *n*, the mean value and the variance of the AUC. As can be seen on Figure 2, the AUC reaches a plateau (0.866, the difference with AUC with 1 repetition is significant) when the number of runs is higher than 30, and the variance is then less than 10^−9^.

Based on this result, GA runs will be repeated 30 times in the following.

### Classifiers

The precision, recall and accuracy have been computed for each of the chosen classifiers. Three of them (LK, PK and QK4) have precision lower than 0.5, meaning that their predictions are even worse than random. Consequently these three classifiers were discarded. The values obtained for the remaining 12 classifiers are given in Table 2. The results obtained show that the different classifiers have very good accuracies. This result is largely due to the fact that the number of positive examples is about ten times lower than the number of negative examples. Consequently, a classifier which predicts all candidates as negative would have an accuracy of 0.9, but a precision of 0 and a recall of 0 for the positive examples. SVM-RBF has a precision of 1, showing that this classifier does not give any false positives, however, the recall is only 0.606, which means that it misses 40% of the positives. Apart from SVM-RBF, all classifiers have relatively low precision and recall.

**Table 2:** Precision, recall and accuracy of the retained classifiers.

Classifier	Precision	Recall	Accuracy
SVM-RBF	1.000	0.606	0.975
PART-M2	0.777	0.737	0.970
J48-M2	0.704	0.697	0.963
JRIP-N10	0.665	0.520	0.954
JRIP-N2	0.65	0.591	0.955
PART-M10	0.645	0.561	0.953
PART-M5	0.642	0.626	0.955
SVM-Q2	0.64	0.727	0.958
J48-M5	0.630	0.586	0.953
JRIP-N5	0.615	0.566	0.951
Logistic	0.607	0.414	0.947
J48-M10	0.564	0.465	0.944

Classifiers have been trained on the same learning set as the genetic algorithm, in 10-fold cross-validation.

**Figure 3:**
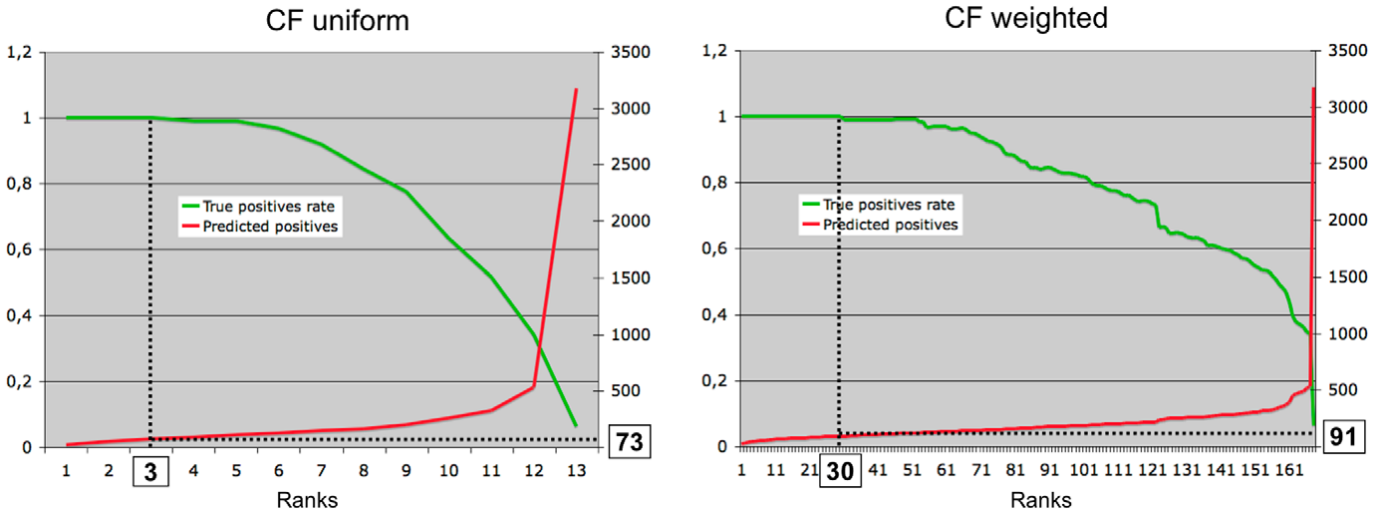
True positive rate for uniform and weighted collaborative filtering. The true positive rate (green) and the total number of positives are plotted for uniform (left) and weighted (right) collaborative filtering, as a function of the category. The vertical and horizontal dotted lines give the category, and the corresponding number of conformations predicted as positives, above which the true positive rate decreases under 1.

The different classifiers have first been combined using an uniform collaborative filtering scheme. In this configuration, each classifier votes for each conformation. Its vote can be *positive* or *negative*. Consequently, a given conformation can receive from 12 to 0 positive votes. Thus, 13 different groups are created, which can be ordered by decreasing numbers of positive votes. When applied to the learning set in 10-fold cross-validation, the three best categories (13, 12, and 11 positive votes) contain only native conformations (Figure 3). This means that the 73 best ranked conformations are true positives.

However, when considering thousands of conformations, 13 categories are not sufficient for efficiently ranking, since many non-equivalent conformations have the same rank (granularity 0.05). To address this problem, we have used an averaged voting protocol (weighted collaborative filtering). Each classifier still votes “positive” or “negative” for each conformation, but the vote is weighted by the precision of the classifier. Since the 12 precisions are all different, the votes of the different classifiers are not equivalent anymore, which results in 2^12^ = 4096 different categories. Consequently, conformations can be classified in 4096 categories, which can be ranked as a function of their positive score (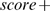, see Methods). Again, the best categories contain only true positives (see Figure 3). The results are even better than those obtained with uniform CF, since the first non-native conformation belongs to category 31, which means that the 91 best ranked conformations are natives.

However, when considering millions of conformations, 4096 categories are still not sufficient (granularity 0.15). For example, when using the weighted-CF method on the learning set, the best category (only positive votes) contains 24 conformations. Consequently, this method cannot be used for ranking large data sets.

### Combination of collaborative filtering and genetic algorithm

**Figure 4:**
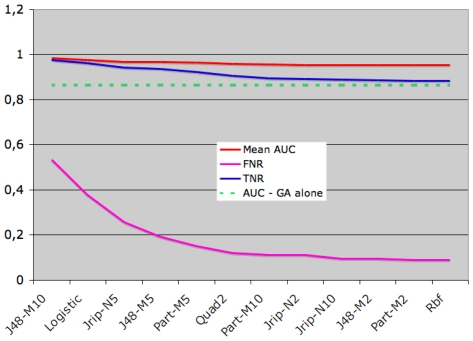
Evolution of AUC, true negative rate (TNR) and false negative rate (FNR) in CF-GA using increasing number of classifiers. Classifiers were added to the collaborative filter, using averaged voting, in increasing precision order. For example, abscissa “JRIP-N5” corresponds to the CF-GA method using J48-M10, Logistic and JRIP-N5 classifiers. Green and red curves correspond to AUC of GA method (which is constant since it doesn't use the classifiers, shown for comparison) and CF-GA method respectively. TNR: true negative rate; FNR: false negative rate.

Since CF efficiently eliminates non-native conformations, we have used CF to weight the GA score (see Methods). This is what we call the collaborative filtering - genetic algorithm (CF-GA) method. The averaged voting configuration was used, and the CF-GA score is obtained by multiplying the GA score by the ratio of the exponential of positive and negative CF scores. Consequently, the score of conformations classified as negatives by a majority of classifiers have very low CF-GA scores. Figure 4 shows the evolutions of AUC, true negative rate (TNR) and false negative rate (FNR) as we add more classifiers in the CF (in increasing precision order).

Another way of combining the two methods is to: first classify the candidate conformations using the CF, retain only the candidates of the best classes, then use the GA to rank them. To evaluate this approach, we retained all the candidate conformations which rank was lower than 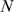 (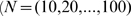 have been tested). These were then ranked using GA. The best results have been obtained with 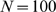, but this method proved less efficient than the CF-GA (see *CF then GA* in Table 3).

**Table 3:** Evaluation of the CF-GA method.

Target		With RMSD filtering												No RMSD filtering							
		GA				CF			CF-GA					CF – GA					CF then GA		
		best		N	R	best	N	R	best		N		R	best		N		R	best	N	R
*fnat* criterion only																					
T22		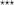		3	4	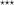	2	3	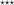		2		4	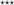	2		4		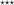	2	4
T23		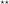		10	1	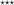	9	1	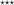		9		1	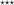	10		1		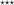	10	1
T25				1	5	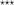	2	2	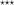		2		2	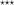	4		2		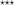	4	2
T26				2	6	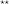	5	1	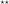		5		1	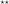	6		1		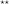	7	1
T27		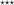		3	6	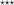	5	1	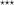		4		1	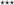	5		1		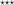	6	1
T29		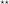		2	5	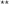	4	1	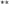		2		2	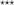	6		2		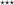	6	2
T32				2	3	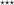	2	3	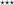		3		10		2		6		-	0	16
T35		-		0	-		1	2			1		1		1		1		-	0	-
T37				1	1		1	5			1		3		1		3		-	0	16
T39		-		0	107	-	0	48	-		0		99	-	0		205		-	0	-
T40A		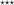		2	2	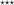	2	1	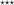		3		1	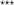	4		1		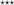	5	1
T40B		-		0	13	-	0	13	-		0		16	-	0		48		-	0	40
All *CAPRI* criteria																					
T25	-		0		13		1	5			1	5			2		6			2	6
T29			1		8		2	1			2	2		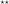	6		2		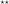	6	1
T32	-		0		36	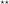	1	3		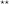	1	10		-	0		18		-	0	16
T35	-		0		NA	-	0	NA		-	0	NA		-	0		167		-	0	-
T37	-		0		14	-	0	13		-	0	17		-	0		18		-	0	18
T39	-		0		NA	-	0	NA		-	0	NA		-	0		652		-	0	-
T40A	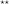		1		3		1	1			1	1		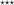	5		1		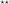	4	1
T40B	-		0		28	-	0	13		-	0	16		-	0		48		-	0	33

Best quality conformation found in the top 10 ranked solutions from target 22 to target 40 for genetic algorithm (GA), collaborative filtering (CF) and combination of the previous two (CF-GA) methods, with RMSD filtering. Same results are given for the CF-GA method without RMSD filtering, and for the *CF then GA* method. N: Numbers of acceptable or better solutions in the top 10; R: rank of the first acceptable or better solution for each target. Numbers of high quality (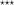), medium quality (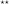), acceptable (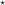) and incorrect conformations in each ensemble and for each method when using RMSD filtering are given in Table 4.

**Table 4:** Total number of conformations is each category before and after RMSD filtering, using 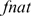 criterion.

	GA						CF				CF-GA					Without RMSD filtering				
Target	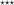	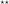				I	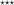	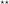		I	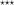	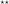		I		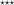	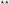			I
*fnat* criterion only																				
T22	12	14		6		40	12	13	7	40	12	13	8		36	32	29		98	113
T23	4	10		23		12	4	10	23	12	2	10	24		13	24	36		189	37
T25	1	0		2		42	1	0	2	38	1	0	2		2	13	2		13	88
T26	12	4		21		165	10	8	21	166	9	7	20		167	537	33		106	641
T27	29	39		39		186	34	36	40	183	32	37	40		192	399	131		106	654
T29	0	3		0		67	0	2	12	60	1	1	16		58	62	78		59	163
T32	1	0		8		171	1	0	7	172	0	1	9		172	1	11		184	376
T35	0	0		3		168	0	0	2	157	0	0	1		159	0	0		8	491
T37	2	2		23		339	1	3	24	337	4	0	21		347	45	34		119	1497
T39	1	1		5		325	0	1	6	324	0	1	5		321	4	1		20	1275
T40A	1	0		7		247	1	0	7	244	1	0	5		248	366	36		119	1439
T40B	2	1		2		249	2	0	1	249	2	1	0		186	165	22		72	1701
All *CAPRI* criteria																				
T25	0		0		1	44	0	0	1	40	0	1	0		44	0	6		14	96
T29	0		0		3	67	0	0	2	72	0	1	2		73	1	76		66	219
T32	0		1		0	179	0	1	0	180	0	0	1		181	0	3		12	557
T35	0		0		0	161	0	0	0	159	0	0	0		160	0	0		3	496
T37	0		3		3	360	0	2	3	360	0	3	2		367	11	46		42	1596
T39	0		1		0	371	0	0	0	331	0	0	0		327	0	3		1	1296
T40A	0		0		2	252	0	0	2	250	0	1	0		253	90	151		150	1569
T40B	2		0		0	252	2	0	0	250	1	1	0		187	102	54		30	1774

Numbers of high quality (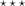), medium quality (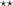), acceptable (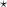) and incorrect (I) conformations in the CAPRI scoring ensembles for each target using 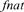 criterion only or all CAPRI criteria, with and without RMSD filtering.

Using the 12 classifiers, the AUC is 0.98, but more importantly, the FNR is only 0.09, meaning that more than 90% of the conformations classified as natives are indeed natives. Unlike collaborative filtering (CF), the GA method gives unique ranks for all conformations (granularity 1). Using the CF-GA method, the global granularity is lower, mostly because conformations classified as non-natives by a majority of classifiers have very few different, but very high, ranks. However, the scores obtained by the 100 best ranked conformations are almost always unique (granularity 0.995), which allows an efficient sorting of the best conformations.

Finally, our tests have shown that similar conformations have a tendency to have very close ranks. To obtain as much diversity as possible in the best ranked solutions, we removed this redundancy using the RMSD between the conformations. A conformation is kept only if its RMSD with better ranked conformations is higher than 5 Å.

Analysis of the most informative parameters in CF and GA allows to better understand the complementarity of the two methods. Indeed, whereas in GA the most informative parameters measure properties of individual residues, CF relies mostly on parameters relative to contacts at the interface. Interestingly, the distance between small amino acids (AGSTCP) appears as the most discriminating parameters in 9 of the 10 analysed filters (the two SVM filters have been excluded). 5 other distances appear in the 10 most discriminating parameters for CF: Hydrophobic-Small, Polar-Positive, Hydrophobic-Negative, Negative-Negative and Polar-Small. The remaining 4 parameters are frequencies of pairs: Hydrophobic-Negative, Polar-Positive, Hydrophobic-Hydrophobic and Polar-Negative. Among the 10 most informative parameters in GA, 7 are relative to individual residues: volumes of R, E, K, P and I; and frequencies of K and 2. The surface of the interface appears in 4th position, and only 2 parameters are relative to contacts at the interface: frequency of Hydrophobic-Polar pairs and distances between Hydrophobic amino acids in contact.

### Ranking of CAPRI ensembles

The CF-GA ranking was applied to CAPRI targets, which were excluded from the learning set. Since no acceptable or better solutions was present in the scoring ensembles for targets 24, 36 and 38, these targets were removed of the analysis.

In a first evaluation, we have used only the 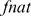 (fraction of native contacts, see Methods) as a quality measure for all structures in the different scoring ensembles. As explained in the Methods section, CAPRI evaluators do consider the 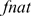 criterion, but also 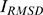 and 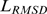 which are different and complementary measures of the distance between the proposed conformation and the native structure. We were unable to reproduce faithfully these measures since they require manual modifications of both the proposed conformation and the native structure (see Methods). Only for targets T25, T29, T32, T35, T37, T39 and T40 were these measures available from the CAPRI website. Consequently, although the 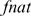 indicator is less stringent than the criteria used by CAPRI evaluators, all targets have been analysed using solely the 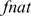 criterion. In parallel, for those targets for which they are available, an evaluation using all CAPRI criteria has been conducted.

We first evaluated the ability of our scoring method to find the native structure within the scoring ensemble. For each target, the native structure was introduced in the scoring ensemble. We were able to rank the native solution in the top 10 for 5 out of 12 targets, and in the top 100 for 9 out of 12 targets.

Our next test was to rank the conformations in the CAPRI ensembles, and count the number of acceptable or better solutions in the top 10. Table 3 shows the results obtained using GA, CF and CF-GA. Numbers of high quality 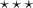), medium quality (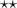), acceptable (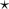) and incorrect conformations in each ensemble and for each method when using RMSD Filtering are given in Table 4.

As can be seen in Table 3, CF-GA is able to rank at least one acceptable or better solution in the top 10 for 10 out of 12 targets. The rank of the first acceptable or better solution is even lower than 4 for 9 targets, and medium quality or better for 8 targets (it should be noted however that for target 35 only acceptable or incorrect conformations were present in the ensemble). When considering all of the CAPRI criteria, CF-GA ranks acceptable or better solutions in the top 10 for 4 out of 8 targets. Interestingly, there seems to be no correlation between our ability to rank the native solution in the top 10 and our ability to ranked an acceptable or better solution in the top 10. Indeed, for targets T22, T26, T27, T29 and T40_A, the native structure is not ranked in the top 10 (even not in the top 100 for T27 and T29), but acceptable or better conformations are found.

CF and CF-GA give very similar results. The best quality conformations and numbers of acceptable or better solutions found in the top 10 are equivalent. However, the average rank of the first acceptable conformation is lower for CF than for CF-GA (3 *vs.* 3.81; target 39 was excluded from this computation since we considered that the ranks obtained were too high to be significant).

When not using RMSD filtering, the use of the 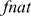 criterion doesn't affect CF-GA global performance. However, using all CAPRI criteria, CF-GA ranks an acceptable or better conformation in the top 10 for only 3 targets out of 8. For target 32, the high quality solution that is found at rank 10 with RMSD filtering, appears at rank 18 without RMSD filtering. More generally, results in Table 3 also show that using RMSD filtering decreases the mean rank of the first acceptable or better solution (3.81 *vs.* 6.36, excluding target 39), but also decreases the mean number of acceptable or better solutions in the top 10 (2.67 *vs.* 3.42, including target 39).

To further evaluate these methods, the enrichment in acceptable or better solutions in the 20% best ranked and 20% worst ranked conformations were computed. Results (Table 5) clearly show that the top 20% is largely enriched in acceptable or better solutions, and even more in medium or better solutions when considering the 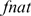 criterion. The comparison between these two categories is more difficult when using all of the CAPRI criteria, since in most cases the computation cannot be made. It can also be seen that CF-GA is better at enriching the top 20% in acceptable or better solutions. It should also be noted that for the three methods, using CAPRI criteria, no acceptable or better solution is ranked in the worst 20%.

**Table 5:** Enrichment in acceptable or better solutions.

	CF-GA				CF				GA			
Target	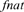		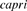		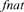		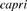		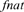		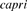	
	E 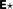	E 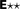	E 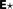	E 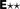	E 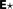	E 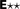	E 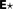	E 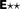	E 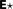	E 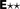	E 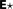	E 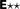
20% best ranked conformations												
T22	*0.6*	0.74	-	-	*0.45*	*0.58*	-	-	*0.56*	0.74	-	-
T23	1.19	1.75	-	-	1.19	1.75	-	-	1.23	1.63	-	-
T25	3.33	5	3	NA	3.04	4.56	4.56	NA	1.67	*0*	*0*	*0*
T26	1.5	1.26	-	-	1.58	1.71	-	-	1.41	0.95	-	-
T27	1.1	1.22	-	-	1.07	1.12	-	-	1.1	1.1	-	-
T29	6.67	5	5	NA	1.89	2.64	5.29	NA	1.21	2.71	1.81	*0*
T32	2.78	5	5	5	2.5	5	5.03	5.03	2.53	5.06	5.06	NA
T35	1.78	NA	NA	NA	2.48	NA	NA	NA	*0*	NA	NA	NA
T37	1.86	2.51	3.34	3.34	1.25	2.5	3	2.5	2.04	3.82	4.08	3.4
T39	*0*	*0*	*0*	*0*	0.72	*0*	NA	NA	*0*	*0*	NA	NA
T40A	3.75	5	4.98	NA	3.71	4.94	4.94	NA	3.32	4.98	4.98	4.98
T40B	1.99	3.32	4.98	4.98	3.29	4.94	4.94	4.94	3.71	3.71	3.71	1.85
**Average**	**2.21**	**2.8**	**4.04**	**3.33**	**1.93**	**2.7**	**4.62**	**4.16**	**1.56**	**2.24**	**3.27**	**2.05**
20% worst ranked conformations												
T22	1.65	1.85	-	-	1.2	1.34	-	-	1.53	1.66	-	-
T23	*0.66*	0.7	-	-	*0.66*	0.7	-	-	1.09	1.23	-	-
T25	*0*	*0*	*0*	NA	1.52	*0*	*0*	NA	*0*	*0*	*0*	*0*
T26	0.82	0.63	-	-	0.92	0.57	-	-	1.13	1.9	-	-
T27	0.91	0.79	-	-	0.93	0.91	-	-	0.92	0.73	-	-
T29	3.33	*0*	*0*	NA	*0.38*	*0*	*0*	NA	1.51	*0*	*0*	*0*
T32	*0*	*0*	*0*	*0*	*0*	*0*	*0*	*0*	*0*	*0*	*0*	NA
T35	1.78	NA	*0*	NA	*0*	NA	*0*	NA	*0*	NA	*0*	NA
T37	0.56	*0*	*0*	*0*	0.54	*0*	*0*	*0*	*0*	*0*	*0*	*0*
T39	1.44	*0*	*0*	*0*	2.15	*0*	*0*	NA	*0*	*0*	*0*	NA
T40A	*0*	*0*	*0*	NA	*0*	*0*	*0*	NA	*0*	*0*	*0*	*0*
T40B	*0*	*0*	*0*	*0*	*0*	*0*	*0*	*0*	*0*	*0*	*0*	*0*
**Average**	**0.93**	**0.36**	**0**	**0**	**0.69**	**0.32**	**0**	**0**	**0.51**	**0.5**	**0**	**0**

The enrichment in acceptable or better conformations (E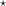) is computed as the proportion of such conformations in the 20% best ranked conformations (respectively worst ranked conformations) divided by the proportion of such conformations is the complete set. Same computation for medium quality or better conformations (E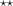). These enrichments are computed using either 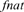 or CAPRI criteria (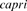), and for the three methods (GA: genetic algorithm, CF: collaborative filtering, CF-GA: hybrid method). Values in italic are not statistically significant.

We have compared these results with the ones obtained by other scoring groups on the 12 targets. As can be seen from Table 6, two of the targets for which we do not find an acceptable or better solution in the top 10 (T35 with all CAPRI criteria, and T39 with either quality measures) were difficult targets, and only one group obtained an acceptable solution for T35, none for T39. It should also be noted that target 35 is not a biological complex, but the assembly of two different modules belonging to the same protein chain.

**Table 6:** Best conformation present in the top 10 for different scoring groups.

Groups	T22	T23	T25	T26	T27	T29	T32	T35	T37	T39	T40A	T40B
C Wang	0	0	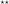	0	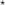	0	0	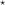	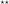	0	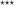	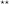
A.M.J.J Bonvin	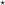	0	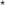	-	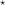	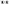	0	0	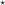	0	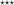	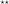
H. Wolfson	-	-	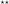	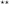	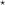	0	0	0	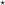	0	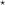	0
P. A. Bates	-	-	-	-	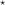	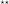	0	0	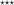	0	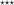	0
Z. Weng	-	-	-	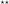	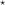	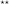	0	0	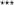	0	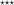	0
J. F.-Recio	-	-	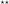	-	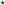	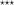	0	0	0	0	0	0
X. Zou	-	-	-	-	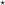	-	0	0	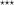	0	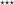	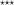
T. Haliloglu	-	-	-	-	-	-	-	-	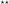	0	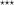	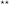
C. J. Camacho	-	-	-	-	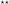	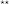	-	-	-	-	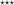	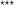
M. Takeda-Shitaka	-	-	-	0	0	0	0	0	-	-	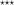	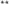
I. Vakser	-	-	-	-	-	-	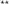	0	0	0	-	-
CF-GA Method	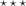 a	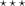 a	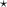	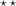 a	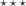 a	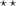	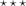	0	0	0	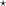	0

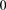
: no acceptable or better solution found, -: group has not participated,

a : 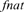 evaluation.

Target 37 was found by most scorers. Our failure for this target is probably related to the fact that this complex is made of three protein chains (A, C and D), and the docking was conducted using only two of these chains. The resulting candidate interfaces, since they represent only a portion of the native interface, are two small to be favourably ranked by our method. Target 40 is also a trimer (chains A, B and C), but this time with two distinct interfaces (CA: target 40A, and CB: target 40B). The GA-CF method successfully finds the CA interface, but fails to favourably rank a good conformation for interface CB. The CA interface is significantly larger than CB (1009.5 *Å^2^ vs.* 731.3 *Å^2^*). Here again, the size of this second interface is two small for our method, especially since much larger interfaces (corresponding to the CA interface) are found in the proposed conformations.

**Figure 5:**
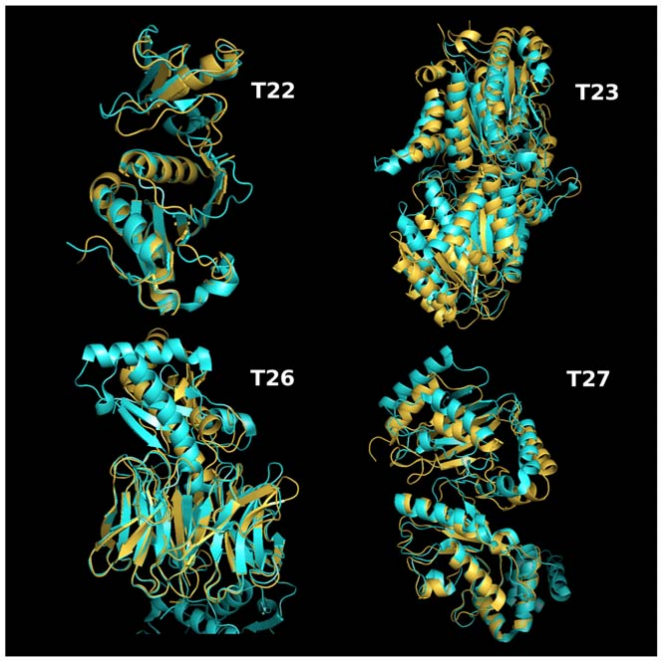
Conformations retained for targets 22, 23, 26 and 27. Native structure in orange, prediction in blue.

For targets 22, 23, 26 and 27, the CAPRI criteria for all proposed conformations are not available. We have compared the categories given to the different conformations by the two criteria sets. Results shown Table 7 show that 99.4% of the conformations evaluated as high quality using the 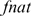 criterion are evaluated as at least acceptable using all criteria (76.8% are even evaluated as medium or better), and 84.7% of the conformations evaluated as medium using the 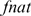 criterion are evaluated as acceptable or better using CAPRI criteria. Consequently, the solutions found in the top 10 for targets 22, 23, 26 and 27 would very likely be considered as acceptable or better using CAPRI criteria. The conformations retained for targets 22, 23, 26 and 27 have 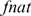 values of 0.95, 0.61, 0.45 and 1 respectively. Upon visual inspection (see Figure 5), and global RMSD computation, we estimated that their CAPRI status would be high, medium, acceptable and high respectively.

**Table 7:** Comparison between 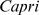 and 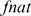 evaluations.

	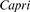				Total
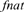	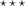	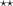	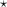	Incorrect	
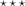	204	298	148	4	654
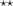		21	106	23	150
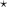			44	547	591
Incorrect				7069	7069
**Total**	204	319	298	7643	8464

For all the conformations in the CAPRI scoring ensembles, the classifications as high-quality, medium-quality, acceptable or incorrect conformation using only 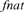, or complete CAPRI are compared. For example, there are 298 conformations classified as medium-quality using CAPRI criteria and high-quality by 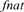 criterion.

Apart from the results obtained by our scoring function, this study shows that the 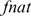 criterion, although and because it is less stringent than the CAPRI criteria, allows a better estimation of the performances of prediction methods. Indeed, predictions that correctly identify the interface area on both protein would be considered *incorrect* using the CAPRI criteria, but *acceptable* using the 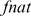 criterion. For predictions having correct contacts, classified as *high* with the 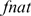, the CAPRI criteria often classifies them as *medium* or even *low*, mostly because of errors in global relative orientations of the two partners. Consequently, the *incorrect* class with the CAPRI criteria doesn't distinguish between these predictions, which have a very high biological utility, and predictions having few native contacts, which are *biologically* wrong. Thus it appears that, from the biologist's point of view, the 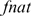 criterion is certainly more useful.

Globally the CF-GA method performs very well, ranking acceptable or better solutions in the top 10 for 8 out of 12 targets. The comparison with other methods is very difficult, since the other methods are evolving and the different groups have not participated to the same rounds. However, it can be seen that the performances of CF-GA compare favorably with current well-performing techniques.

### Conclusion

We have shown that the use of a collaborative filtering strategy combined to a learning procedure leads to an efficient method. Using this technique, we are able to rank at least one acceptable or better solution in top 10 for 10 out of 12 CAPRI targets using solely the 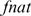 criterion, and 4 out of 8 when using all CAPRI criteria, in cases where scoring ensembles contain acceptable or better solutions. We have also shown that the set of 20% best ranked conformations is largely enriched in medium or better conformations, whereas the set of 20% worst ranked solutions contains very few good models.

The use of RMSD-filtering allows to increase the diversity of the conformations present in the top 10, which decreases the mean rank of the first acceptable or better conformation, but also decreases the number of acceptable or better conformations in the top 10. This is an advantage in an exploration perspective, since the proposed conformations are very different from each other. But this is also a disadvantage in an optimization or refinement perspective, since, for example, a very favourably ranked medium quality conformation can eliminate a high quality conformation having a slightly higher rank.

Finally, we have seen that our method fails on trimers. In the case of target 40 this is largely due to the fact that our method searches the best interface, and is not trained to look for multiple interfaces. Finding these interfaces would probably require training the method specifically on complexes with more than two chains.
